# Response Surface Methodology Optimization of an Acidic Protease Produced by *Penicillium bilaiae* Isolate TDPEF30, a Newly Recovered Endophytic Fungus from Healthy Roots of Date Palm Trees (*Phoenix dactylifera* L.)

**DOI:** 10.3390/microorganisms7030074

**Published:** 2019-03-08

**Authors:** Fedia Ben Mefteh, Fakher Frikha, Amal Daoud, Ali Chenari Bouket, Lenka Luptakova, Faizah N. Alenezi, Bader S. Al-Anzi, Tomasz Oszako, Neji Gharsallah, Lassaad Belbahri

**Affiliations:** 1 NextBiotech, 98 Rue Ali Belhouane, Agareb 3030, Tunisia; fedia.benmefteh@gmail.com (F.B.M.); ali.chenari.bouket@hotmail.com (A.C.B.); Lenka.Luptakova@uvlf.sk (L.L.); Dr-Faizah@outlook.com (F.N.A.); 2 Faculty of Science, B.P. 1171, 3000, University of Sfax, Sfax 3029, Tunisia; fakher.frikha@fss.rnu.tn (F.F.); amal.daoud42@gmail.com (A.D.); Neji.Gharsallah@fss.rnu.tn (N.G.); 3 Department of Biology and Genetics, Institute of Biology, Zoology & Radiobiology, University of Veterinary Medicine and Pharmacy, Komenského 73, 04181 Košice, Slovakia; 4 Department of Environmental Technology Management, College of Life Sciences, Kuwait University, P.O. Box 5969, Safat 13060, Kuwait; bader.alanzi@ku.edu.kw; 5 Department of Forest Protection, Forest Research Institute, 05-090 Raszyn, Poland; T.Oszako@ibles.waw.pl; 6 Laboratory of Soil Biology, University of Neuchatel, 2000 Neuchatel, Switzerland

**Keywords:** Endophytic fungi, *Penicillium bilaiae*, protease, response surface methodology, Box-Behnken

## Abstract

To explore proteolytic activity of endophytic fungi inhabiting date palm roots, a *Penicillium bilaiae* isolate, displaying the highest level of protease production, has been recovered. Response surface methodology (RSM) was applied to optimize culture conditions for protease production by the fungus. Plackett-Burman design allowed for screening of variables effective in protease production. Results indicated that temperature, initial pH and glucose concentration dramatically affect protease yield. These factors were further optimized using a Box-Behnken design and RSM. A combination of initial pH (6.26), temperature (24.5 °C), glucose (13.75 g/L), NaNO_3_ (1.5 g/L), MgSO_4_ (0.2 g/L), KH_2_PO_4_ (0.5 g/L) and KCl (0.5 g/L) were optimum for maximum production of protease. A 1086-fold enhancement of protease production was gained after optimization. Biochemical properties of fungal protease including the effect of pH and temperature on the activity and the stability of proteolytic enzyme were determined. Moreover, the influence of carbon and nitrogen sources, metal ions, detergents as well as enzyme inhibitors was investigated. Our results highlighted that protease of *Penicillium bilaiae* isolate TDPEF30 could be considered as a promising candidate for industrial applications.

## 1. Introduction

Endophytic fungi spend the whole or a part of their life cycle colonizing the internal tissues of host plants, without causing any visible symptoms [1,2]. Therefore, they have potential to produce a broad range of valuable products with a large spectrum of biological activities and promote the growth and resistance of plants against biotic and abiotic stresses including drought, salinity and phytopathogenic agents [3,4]. Among the metabolites required for industrial and pharmaceutical usages, enzymes are currently attracting much attention [5]. There is an increasing demand for new resources of enzymes, including proteases, with various thermo-stabilities and pH profiles suited to different industrial requirements. Proteases are among the most important commercial enzymes used in food processing, silver recovery, diagnostics, detergent industry and pharmaceutical applications [6]. These biological catalysts account for roughly 60% to 65% of the worldwide enzyme market, which allow them to be the leader of the industrial enzymes [7]. Acid proteases among alkaline and/or neutral proteases are widely used in various food industry processes such as cheese and food processing, animal feed, and X-ray films [8].

Currently, several attempts have been made in exploration of new resources of proteases that possess desired criteria for diverse biotechnological applications. Nowadays, microorganisms represent preferred protease resources due to their susceptibility to genetic manipulation and their bioengineering potentials [9]. Moreover, microbial proteases are almost extracellular enzymes which imply that they are directly secreted into the fermentation medium without intervention of complicated extraction processes compared to plant- and animal-derived proteases [10]. Among proteases belonging to microbes, those originating from fungi are the most recommended regarding their high productivity coupled with a low materials cost. Furthermore, fungi are regarded as GRAS (Generally Recognized As Safe) organisms, with the advantage of the safety manipulation of their products, including proteases [11]. In recent years, great number of fungi has been used to produce proteases using different substrates and different approaches. Currently, the application of response surface methodology (RSM) for optimization has increased remarkably thanks to its advantages over the traditional “one-variable-at-a-time” strategy allowing gain of time and materials as well as consideration of interaction between variables to detect the true optimum [12]. The Plackett-Burman design is generally used for detection among a large number of factors as the significant ones that can be considered for further optimization. RSM is a collection of mathematical and technical approaches for searching optimum conditions, predicting the response and checking the adequacy of the model [13]. The experimental design of Box-Behnken was successfully applied in some fields of biotechnology such as the production of laccases [14], xylo-oligosaccharides [15] and wastewater treatment [16].

Despite the considerable data on the production of proteases, no attempt has been conducted on proteases originating from date palm-related endophytic fungi. The present study is therefore designed to optimize the production of protease from endophytic fungi using a statistical design. Using a newly isolated *Penicillium bilaiae* from the internal tissues of adult date palm tree roots, Plackett-Burman and Box Behnken designs were applied to optimize the conditions of production of a promising acid protease suitable for diverse industrial applications.

## 2. Materials and Methods

### 2.1. Isolation of Endophytic Fungi from Healthy Date Palm Roots

Endophytic fungi were isolated from internal tissues of roots of healthy date palms (variety Deglet-Ennour, Nefta, Tunisia), as described by Hallmann et al. [17]. Briefly, samples were initially washed with tap water for 30 min and then surface sterilized in 70% (*v*/*v*) ethanol followed by 3% (*w*/*v*) NaClO treatment for 3 min. The roots were then rinsed several times with sterile distilled water and allowed to surface dry under aseptic conditions. Each root was cut into 0.5–1 cm pieces using a sterile scalpel. Four root pieces were, consequently, placed on plate surface containing potato dextrose agar (PDA) supplemented with 100 μg/mL streptomycin and incubated for 3 to 5 days at 30 °C [18]. After isolation, pure fungal cultures were obtained by sub-culturing single hyphae several times on new PDA plates.

### 2.2. Molecular Identification and Phylogenetic Analysis of the Endophytic Fungi

Mycelial DNA was extracted from pure culture of fungi using a DNA-Easy Plant Mini kit (QIAGEN, Basel, Switzerland) according to the manufacturer’s specifications. The molecular identification was carried out using the protocol described by Belbahri et al. [19]. The universal primers used for the PCR amplification of the ITS rRNA region were ITS1 (5′-TCCGTAGGTGAACCTGCGG-3′) and ITS4 (5′-TCCTCCGCTTATTGATATGC-3′) [20]. Resulting PCR products were sequenced from both sides and consensus sequences were analyzed using BLASTN available from the National Center of Biotechnology Information (NCBI) web site (http://www.ncbi.nlm.nih.gov/blast/Blast.cgi). Identity of endophytic fungi was based on the percentage of homology to sequences available in the database. CLUSTAL W was used for alignment of endophytic fungal sequences and those recovered from the GenBank [21]. Distances were calculated and maximum likelihood (ML) trees were constructed using the PhyML program [22].

### 2.3. Screening of Protease-Producing Endophytic Fungi on Solid Media

The presence of protease activity was assessed using agar plates containing Czapek Dox medium supplemented with 1% (*w*/*v*) skimmed milk powder [23]. The medium was adjusted to pH 7, and poured into plates under aseptic conditions after sterilization. Mycelia from pure culture of fungi were spot inoculated on agar plates and incubated for 5 days at 30 °C. Protease-producing strains were identified by monitoring clear zone formation around them.

### 2.4. Protease Production from the Endophytic Fungus

Protease production was carried out in 300 mL Erlenmeyer flasks with a working volume of 50 mL of Czapek Dox medium containing (g/L): NaNO_3_ 2.5 g, KH_2_PO_4_ 1 g, MgSO_4_·7H_2_O 0.5 g, KCl 0.5 g supplemented with 1% (*w*/*v*) glucose. The medium was sterilized at 121 °C for 20 min. The flasks were inoculated with 10^6^ spores/mL and incubated on a rotary shaker at 150 rpm. Cultures were centrifuged at 8000× *g* for 15 min and the supernatant was used for determination of proteolytic activity. Experiments were performed in triplicate.

### 2.5. Protease Assay

Protease activity was measured by the method developed by Kembhavi et al. [24] using casein as a substrate: 0.5 mL of the enzyme, suitably diluted, was mixed with 0.5 mL of casein (1%) (*w*/*v*) in 100 mM sodium acetate buffer (pH 5.5). After incubation for 10 min at 50 °C, the reaction was stopped by adding 0.5 mL of TCA (trichloroacetic acid). The mixture was kept for 15 min at room temperature and then centrifuged at 10,000× *g* for 15 min to remove the pellet. The absorbance of the soluble fraction was finally measured at 280 nm. A standard curve was established using tyrosine solutions (0–50 mg/L). One unit of protease activity was defined as the amount of enzyme that releases 1 μmol of tyrosine per minute according to standard curve.

### 2.6. Experimental Designs

#### 2.6.1. Plackett-Burman Design

The independent variables of protease production were initial pH, temperature, MgSO_4_, NaNO_3_, KH_2_PO_4_, KCl and glucose. These variables were selected from a preliminary literature review. A Plackett-Burman design was used for multifactor rapid screening to find the most significant independent factors [25,26,27,28]. Then, the seven factors were investigated using the Plackett-Burman design with a first-order polynomial equation. Each factor was examined at low (−1) and high (+1) levels. Eleven variables (including 4 dummy variables) were screened in 15 experimental runs, as shown in Table 1. The fitted first-order model is:

(1) $$Y=\beta_{0}+\sum \beta_{i}x_{i}$$

*Y* is the predicted response, *β*_0_ and *β*_i_ are constant coefficients and *x*_i_ is the coded independent factors.

#### 2.6.2. Box-Behnken Design and Response Surface Analysis

The Box-Behnken design (BBD) of RSM was employed to optimize the three most significant factors (temperature, glucose concentration and initial pH) for enhancing protease production from *Penicillium bilaiae*, screened by Plackett-Burman design [29]. The three independent factors were investigated at three levels (−1, 0, 1) and the experimental design used for study is shown in Table 2. The protease production was fitted using a second order polynomial equation and multiple regression of the data was carried out for obtaining an empirical model related to the most significant factors. The general form of the second-order polynomial equation is:

*Y* = *β*_0_ + Ʃ*β*_i_*x*_i_ + Ʃ*β*_ii_*x*_i_^2^ + Ʃ*β*_ij_*x*_i_*x*_j_ (2)

where *Y* is the predicted response, *x*_i_ and *x*_j_ are independent factors, *β*_0_ is the model intercept, *β*_i_ is the linear coefficient, *β*_ii_ is the quadratic coefficient and *β*_ij_ is the interaction coefficient.

### 2.7. Data Analysis and Software

Design Expert 7.0.0 (trial version, Stat Ease Inc., Minneapolis, MN, USA) was used for experimental design and regression analysis of the experimental data. Analysis of variance (ANOVA) was employed to estimate the statistical parameters.

### 2.8. Effect of Carbon and Nitrogen Sources on Proteolytic Activity

Different sources of carbon and nitrogen were examined for their effect on the production of protease from the endophytic fungus. In total, eight sources of carbon including starch, galactose, sucrose, dextrose, fructose, mannose, xylose and maltose and seven of simple and complex nitrogen sources including casein peptone, ammonium sulfate, urea, casein, yeast extract, KNO_3_ and malt extract were investigated on the basis of the C/N ratio. The medium amended with glucose and NaNO_3_ as sources of carbon and nitrogen, respectively, was considered as control.

### 2.9. Effect of pH and Temperature on Enzyme Activity and Stability

Both optimal pH and stability of proteolytic activity at different pH values were studied. For the determination of optimum pH, the enzyme preparation was experimented at various pH values (3–9) using casein (1%) as a substrate. The pH stability experiments were conducted after pre-incubation of protease containing supernatant in different buffers for 1 h at optimum temperature (25 °C). The following buffers were used: 100 mM glycine-HCl for pH 3, 100 mM sodium acetate for pH 4–6, Tris-HCl for pH 6–8, glycine-NaOH for pH 9. The remaining activity tests were assessed under standard assay conditions.

The optimum temperature for proteolytic activity was evaluated at pH 6 using protease assay at different temperatures values (10–100 °C). The effect of temperature on the stability of the protease was assessed by pre-incubating the aliquots at different temperatures for a fixed time. Afterwards, the residual proteolytic activity was determined using casein as a substrate. The unheated crude enzyme was considered as control.

### 2.10. Effect of Metal Ions, Detergents and Enzyme Inhibitors on Proteolytic Activity

The effect of metal ions was investigated by measuring the residual activity after addition of 5 mM of CuSO_4_, CaCl_2_, FeSO_4_, ZnSO_4_, MnCl_2_, HgCl_2_, H_3_BO_4_ and NaCl. In addition, triton X-100, tween 80 and 3-[(3-cholamidopropyl) dimethylammonio]-1-propane sulfonate (CHAPS) were added separately to the production medium at a rate of 0.1% to evaluate their effect on enzyme activity. In order to study the effect of inhibitors such as β-mercaptoethanol, ethylene diamine tetra-acetic acid (EDTA), phenylmethylsulfonyl fluoride (PMSF) and dithiothreitol (DTT) at a rate of 1 mM, the enzyme preparations were pre-incubated with each inhibitor for 1 h at 4 °C before determination of proteolytic activity. The aliquots without any additive were considered as the 100% control.

## 3. Results

### 3.1. Screening for Proteolytic Activity and Molecular Identification of Penicillium bilaiae TDPEF30

**Figure 1:**
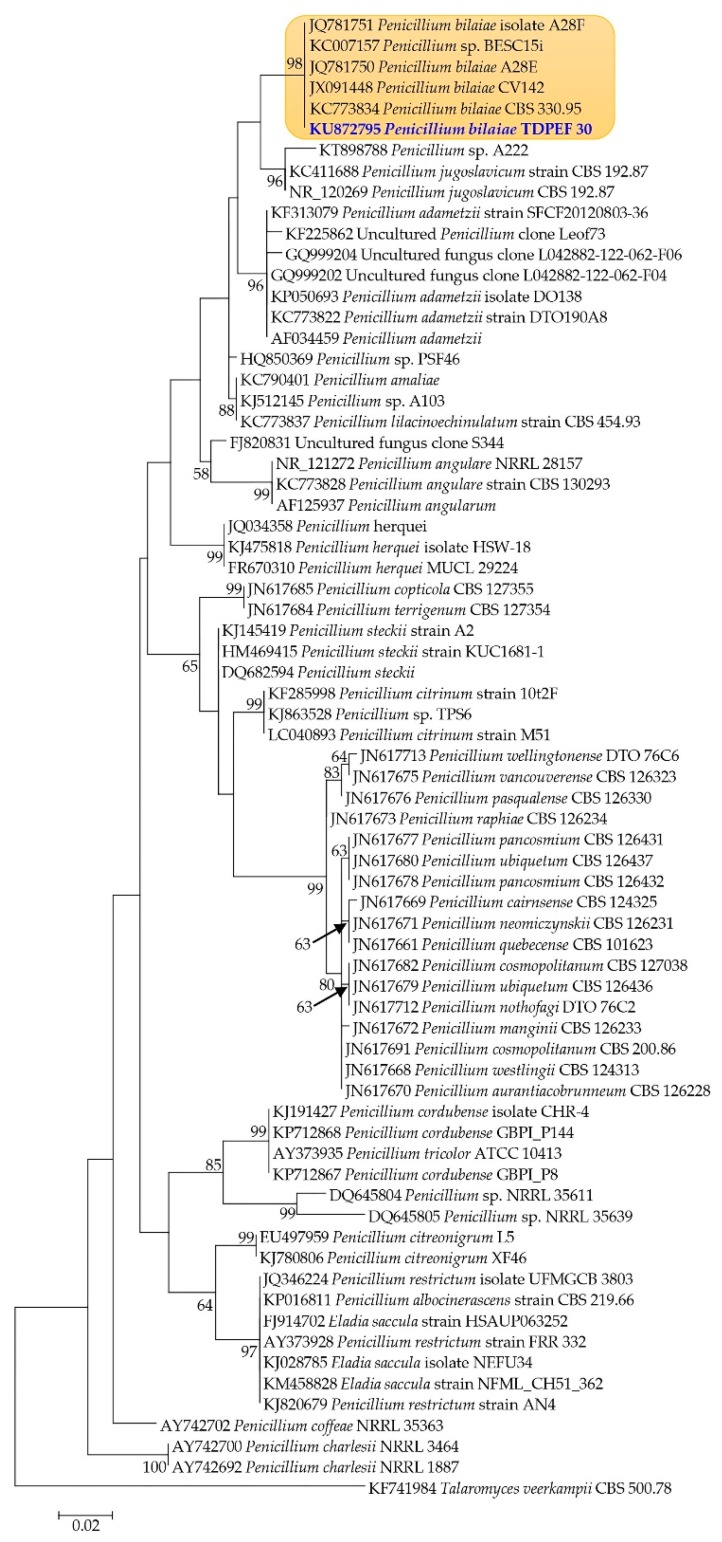
Phylogenetic tree based on 18S rRNA sequence highlighting the phylogenetic position of *Penicillium bilaiae* TDPEF30 within closely related *Penicillium* spp. *Talaromyces veerkampii* (CBS 500.78, KF741984) was used as an outgroup. Bootstrap values are expressed as percentage of 1000 replicates.

In order to explore proteolytic activity of endophytic fungi from date palm roots, a total of 21 fungi were recovered from the internal tissues of adult date palm trees healthy roots (*Phoenix dactylifera* L., Deglet Ennour variety). Recovered fungal isolates were screened on solid medium for their ability to produce proteolytic enzymes (data not shown). Isolate TDPEF30 displayed the highest level of production of protease revealing a halo diameter in the range of 30 ± 0.5 mm. Internal Transcribed Spacer (ITS) regions of fungal rDNA were amplified and sequenced. The identity of isolates was determined based on homology with sequences available in the BLASTN database. TDPEF30 showed high homology to *Penicillium bilaiae* sequences available in the database. Phylogenetic analysis was conducted to ascertain the phylogenetic position and the taxonomy of TDPEF30. As indicated in Figure 1, TDPEF30 lies within *Penicillium bilaiae* isolates with high bootstrap value. Therefore, it has been definitely identified as *Penicillium bilaiae*.

### 3.2. Experimental Designs

#### 3.2.1. Plackett-Burman Design

The Plackett-Burman design was used for screening of the significant factors that affect TDPEF30 protease production. Fifteen experiments were carried out to evaluate the effect of seven factors on the protease production and the results are shown in Table 1. A *t*-test and Fisher’s test were used to identify the significance of each factor on the protease production. Statistical analysis of the response was performed (Table 3). According to the design, variables that have the greatest effect on the production of protease from *Penicillium bilaiae* were glucose concentration, pH and temperature at 95% confidence level. However, pH and the concentration of glucose have positive effects while 4 variables including MgSO_4_, NaNO_3_, KH_2_PO_4_ and KCl concentration have negative effects on the proteolytic activity. Only temperature was found to be significantn in influencing protease activity, as shown in Table 3 (Prob > *F* < 0.05). Non-significant variables with negative effect (MgSO_4_, NaNO_3_, KH_2_PO_4_ and KCl concentrations) were fixed to their low levels corresponding to 0.2, 1.5, 0.5 and 0.5 g/L, respectively. Although glucose concentration and initial pH were not significant at 95% confidence level, they were selected with the temperature for further optimization to obtain a maximal response.

#### 3.2.2. Box Behnken Design (BBD) and Response Surface Analysis

A response surface design was further applied when the optimal region for running the process has been identified [27,28,30]. Based on the Placket-Burman design output, an RSM using the Box Behnken design was applied to determine the optimal levels of the three selected variables (temperature, glucose and initial pH). The respective low and high levels with the coded levels for the three variables are defined in Table 3. A total of 15 experiments with different combinations of the selected parameters were performed. The observed and predicted responses are reported in Table 4. The experimental results were analyzed by standard ANOVA and the BBD was fitted with the second-order polynomial equation:

Ln(*Ŷ*) = 5.798 − 2.194*X*_1_ + 0.157*X*_2_ − 0.101*X*_3_ − 0.565*X*_1_*X*_3_ − 2.305*X*_1_^2^ + 0.428 *X*_2_^2^ − 0.416*X*_3_^2^ (3)

where *X*_1_, *X*_2_ and *X*_3_ correspond to temperature, glucose concentration and pH, respectively.

#### 3.2.3. Analysis of Variance and Validation of the Model

Statistical significance of the model equation was evaluated by the *F*-test for ANOVA. As shown in Table 5, the regression sum of squares was statistically significant when the *F*-test used was at 95% probability level, suggesting that the variation considered by the model was significantly greater than the unexplained variation. The two checkpoint results were employed to validate the fitted model (Table 6). The measured values (*y*_i_) were very close to those calculated (*ŷ*) using the model equation (Table 7). Moreover, the differences between calculated and measured responses were not significant (*t* test, *p* > 0.05). It was, therefore, concluded that the model was adequate in describing the response surfaces, and could be used as a prediction equation in the design space.

#### 3.2.4. Graphical Interpretation of the Response Surface Model

**Figure 2:**
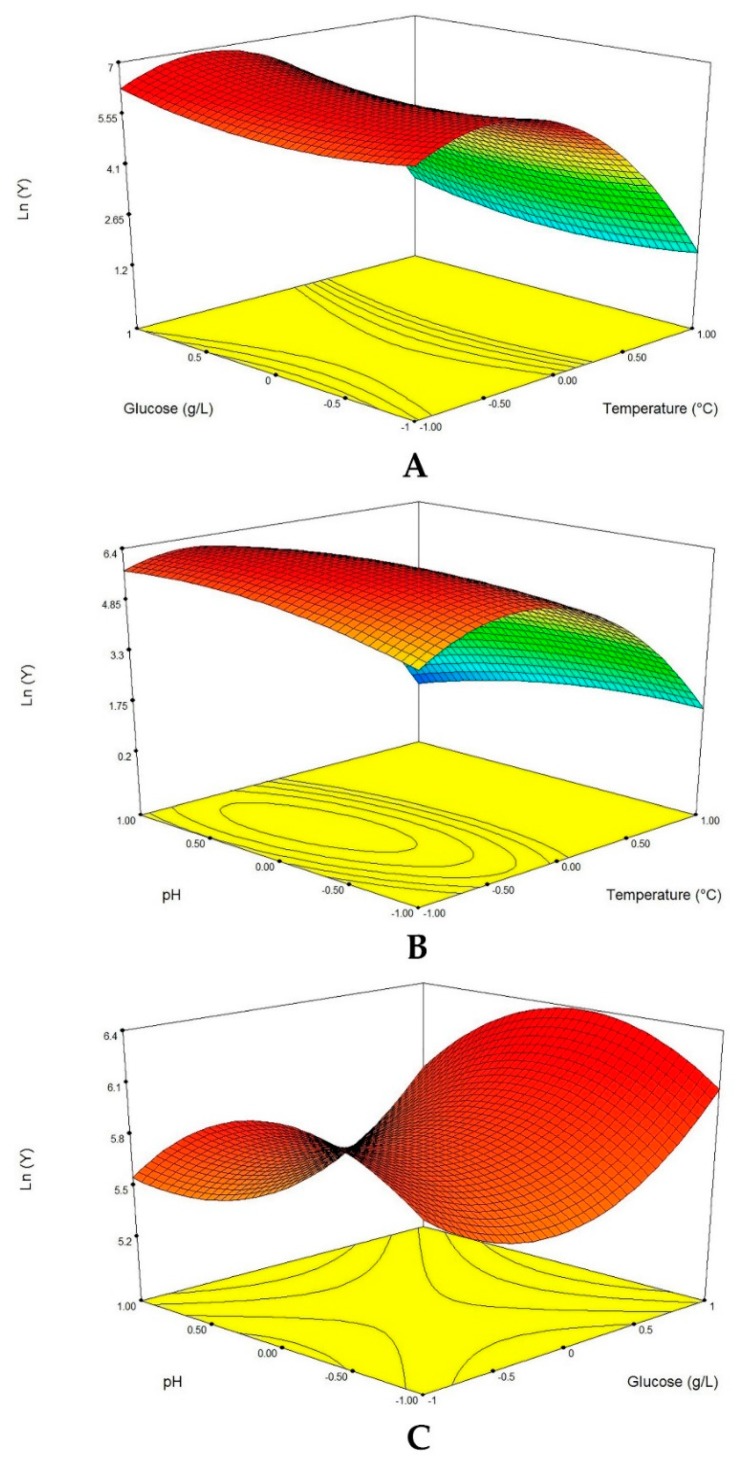
Response surface of protease production from the endophytic fungus *Penicillium bilaiae* showing the interactive effects of the temperature versus glucose concentration (**A**); culture pH versus temperature and glucose (**B**); concentration versus initial pH (**C**).

The protease production was a function of the interaction between the three variables (glucose concentration, pH and temperature). The response surface curves were plotted to explain the interaction of the variables and to determine the optimum level of each variable for maximum response. The response surface curves are shown in Figure 2. Each figure demonstrates the effect of two factors while the other factors were fixed at zero level. The effect of temperature and glucose on the protease production at a fixed pH of 6 is shown in Figure 2A. Decreasing the temperature led to an increase in the protease production, irrespective of the glucose concentration. The response value reached its highest point at 25 °C.

However, at 25 °C, the glucose concentration had no significant impact on the protease production. The effect of temperature and pH on the protease production at a fixed glucose concentration of 11 g/L is presented in Figure 2B. The response value reached its highest point at 25 °C and pH 6. The protease production slightly increases when the pH is equal to 6. Figure 2C shows the effect of glucose concentration and the pH on the protease production at a fixed temperature of 30 °C. Increasing the concentration of glucose resulted in an increase in the response surface. The response value reached its highest point at 14 g/L of glucose concentration and pH 6. Furthermore, the elliptical contour in Figure 2B indicates that an elevation in initial pH as well as the temperature had positive effects on protease activity, while, an increase in the above constituents was found to have negative influence on the protease production from the date palm endophytic fungus (Figure 2A,C). From the surface response graphs and the regression analysis of Equation (3), it could be concluded that the optimal conditions for protease production were located in the region where temperature, initial pH and glucose concentration are in the range of 25 °C, 6 and 14 g/L, respectively.

#### 3.2.5. Effect of Carbon and Nitrogen Sources on Protease Production

**Figure 3:**
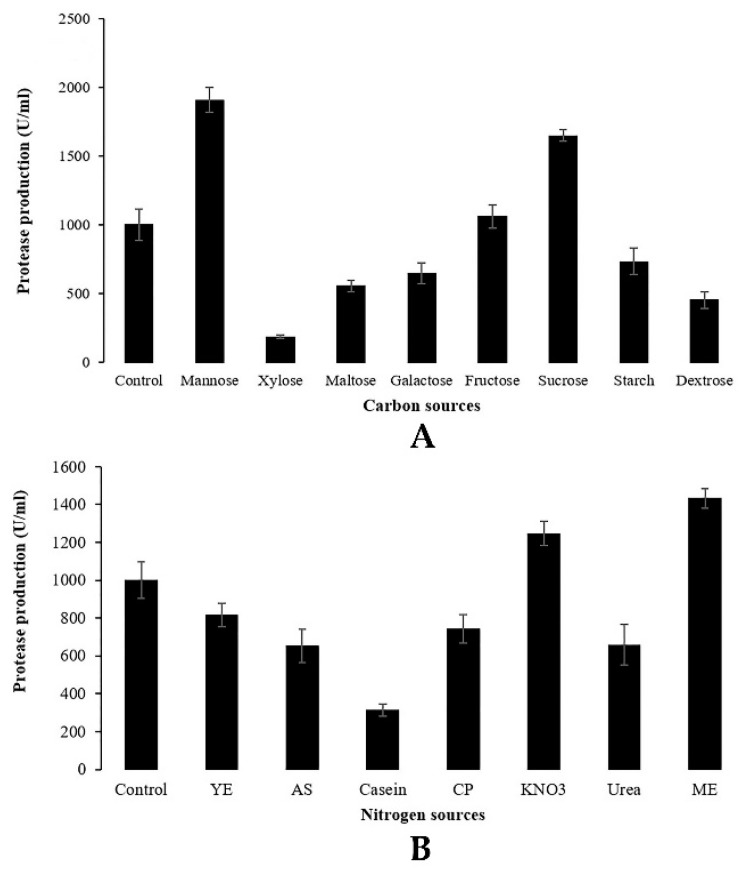
Effect of various sources of carbon (**A**) and nitrogen (**B**) on protease production using C/N ratio. YE yeast extract, AS ammonium sulfate, CP casein peptone, ME malt extract.

A series of experiments were performed to study the effects of various carbon and nitrogen sources on protease production by *Penicillium bilaiae* TDPEF30. Cultures were conducted in flasks containing Czapek Dox medium with respect to the C/N ratio. Results are presented in Figure 3. Among the different sources of examined carbon, maximum protease production was attained in the presence of easily assimilated compounds. Mannose was the best source of carbon for the production of protease (1907.28 U/mL) followed by a complex source of carbon including sucrose (1649.12 U/mL) and fructose (1060.15 U/mL), as shown in Figure 3A.

Both organic and inorganic sources of nitrogen (yeast extract, ammonium sulfate, casein, casein peptone, KNO_3_, urea, malt extract) were added separately to the culture medium to evaluate their influence on protease production. Results reported in Figure 3B clearly indicate that the highest level of protease production was obtained with malt extract (1433.28 U/mL) followed by KNO_3_ (1247.07 U/mL). Among the various sources of carbon and nitrogen tested, mannose and malt extract were found to be the most suitable substrates for the protease production from the endophytic fungus *Penicillium bilaiae* TDPEF30.

#### 3.2.6. Effect of pH and temperature on protease activity and stability

**Figure 4:**
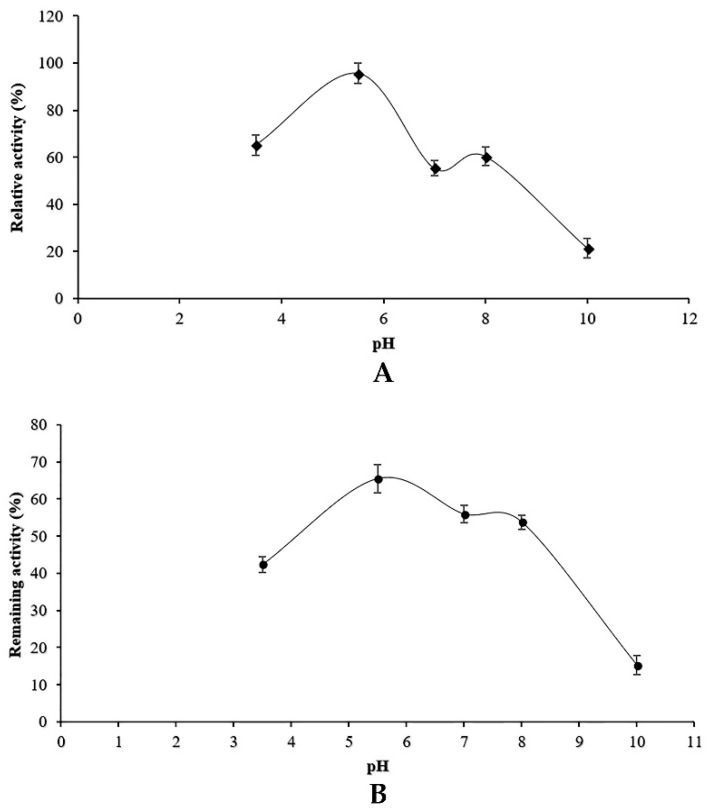
Effect of pH on the activity (**A**) and the stability (**B**) of the proteolytic enzymes of *Penicillium bilaiae*.

The effect of pH on protease activity was determined over a pH range of 3–9 using different buffer solutions. As shown in Figure 4A, maximum protease activity was observed at pH 6, which indicated the acidity of the proteolytic enzyme produced by the endophytic fungus associated with adult date palm trees. The proteolytic enzyme was active at pH 8 since the relative activity at pH 8 was about 50% of that observed at pH 6. Proteases were totally inactivated at pH 9 and 10.

The stability of proteolytic enzymes was evaluated after incubation of enzyme preparation in different buffers with various pH values at 4 °C for 1 h. The results are reported in Figure 4B. Proteases were more stable (>50%) under acidic conditions (pH 5–6.5). The stability decreased at neutral pH and increased slightly at pH 8. The fungal protease was more stable at pH range of 5.5–6.5, as shown in Figure 4B.

**Figure 5:**
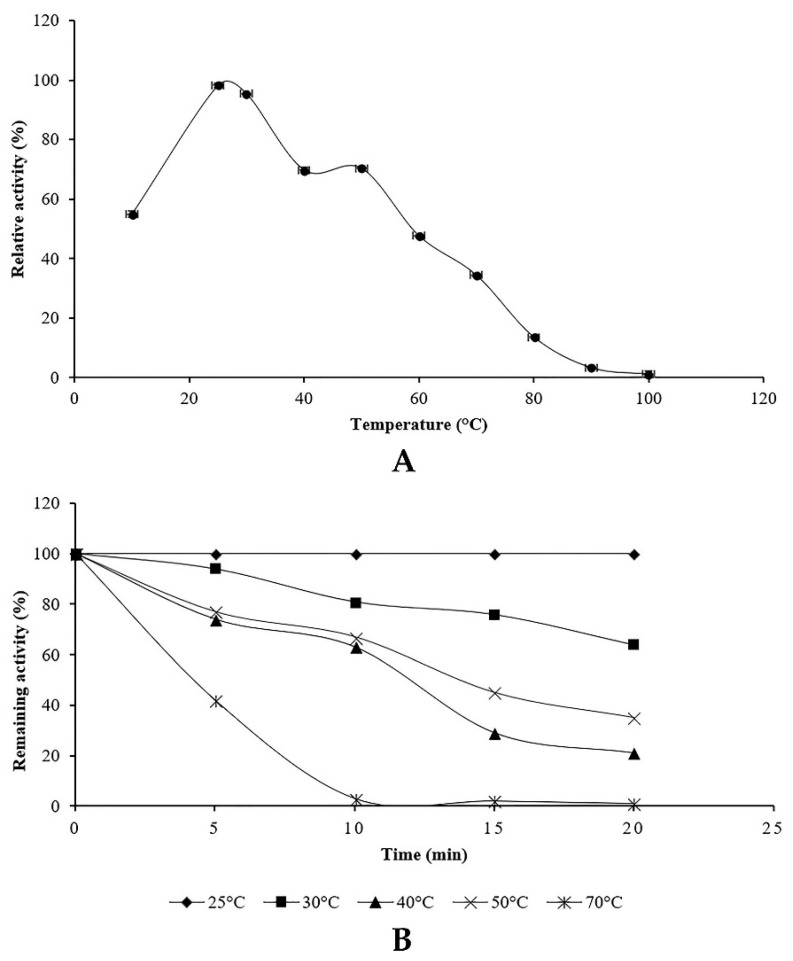
Effect of temperature on the activity (**A**) and the stability (**B**) of proteolytic enzyme of *Penicillium bilaiae*.

Proteolytic enzyme activity of the endophytic fungus *Penicillium bilaiae* TDPEF30 was measured at temperatures ranging from 10 to 100 °C. The relative and the residual activities were determined (Figure 5). The proteolytic activity showed that the enzyme maintained more than 50% of its activity over a range of 20–50 °C with optimum activity at 25 °C using casein as a substrate (Figure 5A).

The effect of temperature on protease stability was evaluated after incubation of crude supernatant at different temperatures ranging from 20 to 70 °C and pH 6 for various incubation periods. As shown in Figure 5B, the enzyme was highly stable at 25 °C. The protease was able to retain 100% of its activity at 25 °C even after 2 h of incubation while it was inactivated by losing 90% of its initial activity after 10 min of incubation at 70 °C.

#### 3.2.7. Effect of Metal Ions, Detergents and Enzyme Inhibitors on Proteolytic Activity

The influence of metal ions such as Cu^2+^, Ca^2+^, Fe^2+^, Zn^2+^, Mn^2+^, Hg^2+^, B^3+^ and Na^2+^ (5 mM), detergents (0.1%), namely, tween 80, CHAPS and triton X-100 as well as enzyme inhibitors (5 mM) β-mercaptoethanol, DTT, EDTA and PMSF were tested at pH 6 and 25 °C on protease activity from the endophytic fungus *Penicillium bilaiae* TDPEF30. The results are reported in Table 8. Concerning the effect of metal ions, the relative activity was measured after addition of 5 mM of the respective ion to the reaction mixture. According to Table 7, the addition of Mn^2+^, B^3+^ enhanced the proteolytic activity by 114 and 191%, respectively, while Cu^2+^, Ca^2+^, Fe^2+^ and Hg^2+^ inhibited the enzyme activity to approximately 14–54%. Furthermore, Zn^2+^ and Na^2+^ had no effect on protease activity.

The effect of detergents on the production of protease from the endophytic fungus *Penicillium bilaiae* TDPEF30 was also investigated. Only CHAPS decreased the enzyme production while Tween 80 and triton X-100 increased the protease activity yield by 181 and 193%, respectively.

Effect of different enzyme inhibitors was studied on protease production by the endophytic fungus *Penicillium bilaiae* TDPEF30. The results illustrated in Table 8 proof that while the fungal protease was completely inhibited by PMSF, β-mercaptoethanol, DTT and EDTA had no effect on enzyme activity.

## 4. Discussion

The improvement of enzyme production of endophytic microorganisms is the purpose of several investigations owing to their abilities to produce many valuables metabolites with potential bioactivity [31]. In the present study, endophytic fungi were isolated from the root internal tissues of adult date palm trees. These isolates were evaluated for their ability to produce proteases. Plating on skim milk agar medium, as described by Saran et al. [32], is an easy and simple technique to screen for protease activity, although it cannot be recommended for quantitative analysis. The wide use of proteases in biotechnological and industrial processes has attracted researchers to obtain proteolytic enzymes that exhibit various properties suitable for different applications. However, each microbial strain has its specific conditions to maximize protease production. According to our results, the endophytic fungus *Penicillium bilaiae* TDPEF30 was found to be the best producer of proteases and was selected for further assays of protease production in liquid medium. Few studies have been done to induce protease production from endophytic fungi using a statistical design. Using Czapek Dox as fermentation medium, the TDPEF30 endophytic strain displayed the best level of protease production with 203 ± 2.1 U/mL. Czapek Dox is an easy to prepare medium with simple components [33].

RSM was carried out to optimize culture medium nutrients and conditions for the production of protease by the endophytic fungus *Penicillium bilaiae* TDPEF30. At the beginning, the significant variables that influence the protease production were selected using Plackett-Burman Design. Rodriguez et al. [34] as well as Hajji et al. [35] reported that proteases from *Penicillium* and *Aspergillus* fungi were affected by temperature and pH. In line with these studies, it was demonstrated in the present study that glucose concentration, pH and temperature had significant effects on protease synthesis by the endophytic fungus *Penicillium bilaiae* TDPEF30. Then, the BBD was adopted to find out the optimum levels of each significant variable and their interactive effects on protease yield.

Nowadays, application of RSM in biotechnology is increasingly used to statistically determine the best condition for enzyme production, owing to its easy applicability, validity and reliability [36]. In our study, protease production by the endophytic fungus of adult Tunisian date palm was enhanced. However, maximum protease production was achieved at temperature 24.46 °C, pH 6.26 and 13.75 g/L glucose while the other components were used at the lowest level 0.2 g/L MgSO_4_, 1.5 g/L NaNO_3_, 0.5 g/L KH_2_PO_4_ and 0.5 g/L KCl. The RSM process resulted in a proteolytic activity of 1086.95 U/mL, with a 5.8 increase compared to 186.21 U/mL obtained before optimization. Furthermore, the high degree of similarity between experimental and predicted values reflected the accuracy and the validity of the experimental design applied.

It is well known that microbial protease production is dependent on the availability of both carbon and nitrogen sources, which play a regulatory role in enzyme synthesis [37]. In the current study, various sources of carbon and nitrogen were investigated in order to find the maximum protease production by the endophytic fungus *Penicillium bilaiae* TDPEF30. The significant effect of sources of carbon and nitrogen either on protease activity or on enzyme production is shown in Figure 3. Concerning carbon source influences, the highest level of protease activity was obtained in presence of the simple carbohydrate mannose with 1907.28 U/mL followed by complex sources of carbon, namely, sucrose and fructose, with 1649.12 and 1060.15 U/mL, respectively. These different carbon sources have the benefits of their commercial availability. Nonetheless, other carbon sources such as xylose, dextrose, maltose, galactose and starch decreased the protease production by the endophytic fungus TDPEF30 compared to the control glucose-based medium. In general, both organic and inorganic sources of nitrogen were evaluated for their influence on protease production. In the present study, only malt extract and KNO_3_ increased the proteolytic enzyme yield compared to the control with NaNO_3_. Our findings are in agreement with the results of Hameed et al. [38], who reported that the addition of mannose induced significant proteolytic activity in etiolated wheat leaves. In addition, Sumantha et al. [39] and Zaferanloo et al. [33] found that protease production by *Rhizopus microsporus* NRRL 3671 and *Alternaria alternata*, respectively, was significantly influenced by specific sources of carbon and nitrogen, which could be explained by the specificity of protease enzyme.

Along with carbon and nitrogen sources, the environmental conditions such as pH medium and temperature could play an important role in the induction or repression of protease activity. Our results indicated that *Penicillium bilaiae* produced protease at pH range 5–6.5 with an optimum at six. In addition, maximum protease activity was obtained at 25 °C. In this study, it was found that protease activity increased slightly at pH 8 and 50 °C. These findings could be explained by the presence of isoenzymes produced by the endophytic fungus TDPEF30 during fermentation. This suggests the potentiality of endophytic fungus TDPEF30 to produce a wide range of valuable enzymes with different properties and even in the same class. Enzyme inactivation represents one of the major limitations in the use of proteases in biotechnological processes. The protease produced by the endophytic fungus TDPEF30 lost its activity at 70 °C and pH 10. Most fungi have an optimum of temperature at the range of 28–30 °C (Hajji et al., 2008; Germano et al., 2003) [35,40]. However, earlier studies reported that production of proteases from *Penicillium* species like *P. citrinum*, *P. perpurogerum* and *P. funculosum* was obtained at temperature lower than 30 °C [41].

The effect of metal ions on protease production by the endophytic fungus *Penicillium bilaiae* TDPEF30 at a concentration of 5 mM was evaluated under optimal conditions of pH and temperature. Results indicated that the addition of both Mn^2+^ and B^3+^ enhanced the fungal protease activity. In contrast, Cu^2+^, Ca^2+^, Fe^2+^ and Hg^2+^ significantly decreased protease activity. Similar effects of Mn^2+^ on proteolytic enzyme production were obtained with *Penicillium* sp. [42]. This finding might be attributed to manganese ion involvement in stabilization of the enzyme molecular structure. In contrast, our findings are in disagreement with the study of Negi & Banerjee [37], who found that Ca^2+^ and Hg^2+^ simulated protease production by *Aspergillus awamori*.

The influence of detergents on protease activity was well studied with bacteria, but with fungi, few studies have been reported. Among the detergents, only CHAPS decreased protease activity. Our results are in agreement with the study of Guleria et al. [43], which revealed that the alkaline protease from *Bacillus amyloliquefaciens* SP1 increased in the presence of triton X-100. Furthermore, Guleria et al. [43] reported the high level of proteolytic activity obtained after incubation of enzyme solution in tween 80.

The effect of various enzyme inhibitors (used at a concentration of 5 mM) was determined to evaluate their influence on protease activity. Only PMSF, a well-known as serine protease inhibitor, inhibited protease activity from the endophytic fungus of adult date palm, TDPEF30. We speculate that the protease optimized during the current study can be classified as serine protease.

## 5. Conclusions

The present study is the first to produce protease from a newly isolated fungus from the roots of adult date palm belonging to the Deglet Ennour variety using a statistical design. Further analysis on Czapek Dox medium revealed that *Penicillium bilaiae* TDPEF30 (GenBank accession for ITS rRNA sequence: KU872795) has the maximum level of protease production. Plackett-Burman design and RSM approaches were employed for optimization of culture and environment conditions and were shown to significantly enhance protease production. The individual and the interactive effects of the three significant factors, namely, temperature, initial pH and glucose concentration were determined. Maximal levels of protease production from the endophytic fungus *Penicillium bilaiae* TDPEF30 were obtained at 24.46 °C, pH 6.26, 13.75 g/L glucose, 0.5 g/L KH_2_PO_4_, 0.5 g/L KCl and 0.2 g/L MgSO_4_. Under these conditions, the *Penicillium bilaiae* TDPEF30 protease activity could reach 1086.95 U/mL. Further studies, such as purification and industrial application of protease from this promising strain, are in progress.

## Figures and Tables

**Table 1:** Plackett-Burman design for screening of significant factors affecting the protease production by *Penicillium bilaiae*.

Run	Initial pH	Temperature (°C)	MgSO_4_ (g/L)	NaNO_3_ (g/L)	KH_2_PO_4_ (g/L)	KCl (g/L)	Glucose (g/L)	Response (U/mL)
1	8	40	0.2	3.5	1.5	1.5	8	56.56
2	4	40	0.8	1.5	1.5	1.5	14	35.40
3	8	20	0.8	3.5	0.5	1.5	14	62.07
4	4	40	0.2	3.5	1.5	0.5	14	38.93
5	4	20	0.8	1.5	1.5	1.5	8	53.81
6	4	20	0.2	3.5	0.5	1.5	14	77.83
7	8	20	0.2	1.5	1.5	0.5	14	21.16
8	8	40	0.2	1.5	0.5	1.5	8	83.58
9	8	40	0.8	1.5	0.5	0.5	14	57.62
10	4	40	0.8	3.5	0.5	0.5	8	41.82
11	8	20	0.8	3.5	1.5	0.5	8	186.21
12	4	20	0.2	1.5	0.5	0.5	8	59.60
13	6	30	0.5	2.5	1	1	11	53.88
14	6	30	0.5	2.5	1	1	11	43.73
15	6	30	0.5	2.5	1	1	11	50.22

**Table 2:** Levels of variables tested in Box Behnken Design (BBD) for optimization of protease production.

Factor	Range and Levels		
	−1	0	1
*X*_1_ Temperature (°C)	20	30	40
*X*_2_ Glucose (g/L)	8	11	14
*X*_3_ pH	4	6	8

**Table 3:** Determination of significant variables for production of protease by the endophytic fungus *Penicillium bilaiae* using Plackett-Burman Design.

Source	Sum of Squares	df	Mean of Square	*F*-Value	Prob > *F*	Contribution %	Effect
Model	9385.037	7	1340.720	4.345	0.047		
A-pH	673.051	1	673.051	2.181	0.190	4.24	14.98
B-Temperature	6325.480	1	6325.480	20.499	0.004	39.81	−45.92
C-MgSO_4_	302.304	1	302.304	0.980	0.361	1.90	−10.04
D-NaNO_3_	75.350	1	75.350	0.244	0.639	0.47	−5.01
E-KH_2_PO_4_	229.425	1	229.425	0.744	0.422	1.44	−8.75
F-KCl	50.225	1	50.225	0.163	0.701	0.32	−4.09
G-Glucose	1729.200	1	1729.200	5.604	0.056	10.88	24.01

**Table 4:** Box Behnken matrix and response results for optimization of protease.

Run	Factors			Protease Activity U/mL	
	*X*_1_ (°C)	*X*_2_ (g/L)	*X*_3_	Experimental	Predicted
1	−1	−1	0	417.57	386.82
2	1	−1	0	6.20	4.80
3	−1	1	0	328.69	529.61
4	1	1	0	7.61	6.58
5	−1	0	−1	144.73	122.41
6	1	0	−1	3.73	4.71
7	−1	0	1	390.76	309.52
8	1	0	1	1.05	1.24
9	0	−1	−1	329.40	315.42
10	0	1	−1	441.55	431.85
11	0	−1	1	177.04	257.53
12	0	1	1	480.34	352.59
13	0	0	0	398.52	329.56
14	0	0	0	292.72	329.56
15	0	0	0	306.83	329.56

**Table 5:** Analysis of variance for surface response.

Source of Variation	Sum of Squares	df	Mean of Square	*F* Value	*p* Value Prob > F
Regression	61.34	7	8.76	78.84	<0.0001 *
Residual	0.78	7	0.11		
Cor Total	62.12	14			

* Statistically significant at 95% of confidence level. Std. Dev. = 0.333, *R*^2^ = 0.987.

**Table 6:** Experimental condition of the check-points.

Run	Variables Values			Response (U/mL)	
	*X*_1_ (°C)	*X*_2_ (g/L)	*X*_3_	Experimental	Predicted
1	24.46	13.75	6.26	1086.95	924.23
2	24.09	13.78	5.59	744.85	842.16

*X*_1_ temperature, *X*_2_ glucose concentration, *X*_3_ initial pH.

**Table 7:** Validation of the model with the check-points.

Run	*y*_i_	*ŷ*_i_	Residual *e* = *y*_i_ − *ŷ*_i_	t.exp.	Laverage (dU)	Signif.%
	6.991	6.830	0.161	0.437	0.224	67.50
	6.613	6.737	−0,124	−0,335	0.235	74.72

*y*_i_ = Ln(*Y*exp.); *ŷ*_i_ = Ln(*Y*calc.).

**Table 8:** Effect of metal ions (5 mM), detergents (0.1%) and enzyme inhibitors (5 mM) on protease activity of the endophytic fungus *Penicillium bilaiae*.

Chemicals	Concentrations	Units	Relative Activity (%)
Control	-	-	100
Cu^2+^	5	mM	21 ± 3
Ca^2+^	5	mM	32 ± 4
Fe^2+^	5	mM	54 ± 1
Zn^2+^	5	mM	100
Mn^2+^	5	mM	114 ± 2
Hg^2+^	5	mM	14 ± 4
B^3+^	5	mM	191 ± 1
Na^2+^	5	mM	100
PMSF	5	mM	0
EDTA	5	mM	100
β-Mercaptoethanol	5	mM	100
DTT	5	mM	100
Tween 80	0.1	%	181 ± 1
CHAPS	0.1	%	83 ± 1
Triton X-100	0.1	%	193 ± 2
